# Two-dimensional nuclear magnetic resonance spectroscopy with a microfluidic diamond quantum sensor

**DOI:** 10.1126/sciadv.aaw7895

**Published:** 2019-07-26

**Authors:** Janis Smits, Joshua T. Damron, Pauli Kehayias, Andrew F. McDowell, Nazanin Mosavian, Ilja Fescenko, Nathaniel Ristoff, Abdelghani Laraoui, Andrey Jarmola, Victor M. Acosta

**Affiliations:** 1 Center for High Technology Materials and Department of Physics and Astronomy, University of New Mexico, Albuquerque, NM 87106, USA.; 2 Laser Center of the University of Latvia, Riga, LV-1586, Latvia.; 3 Sandia National Laboratories, Albuquerque, NM 87123, USA.; 4 NuevoMR LLC, Albuequerque, NM 87106, USA.; 5 Department of Physics, University of California, Berkeley, CA 94720, USA.; 6 ODMR Technologies Inc., El Cerrito, CA 94530, USA.

## Abstract

Quantum sensors based on nitrogen-vacancy centers in diamond have emerged as a promising detection modality for nuclear magnetic resonance (NMR) spectroscopy owing to their micrometer-scale detection volume and noninductive-based detection. A remaining challenge is to realize sufficiently high spectral resolution and concentration sensitivity for multidimensional NMR analysis of picoliter sample volumes. Here, we address this challenge by spatially separating the polarization and detection phases of the experiment in a microfluidic platform. We realize a spectral resolution of 0.65 ± 0.05 Hz, an order-of-magnitude improvement over previous diamond NMR studies. We use the platform to perform two-dimensional correlation spectroscopy of liquid analytes within an effective ∼40-picoliter detection volume. The use of diamond quantum sensors as in-line microfluidic NMR detectors is a major step toward applications in mass-limited chemical analysis and single-cell biology.

## Teaser

We demonstrate a microfluidic platform for multidimensional NMR using prepolarization and detection with a diamond quantum sensor.

## INTRODUCTION

Nuclear magnetic resonance (NMR) spectroscopy is a powerful and well-established method for compositional, structural, and functional analysis used in a wide range of scientific disciplines. Conventional NMR spectrometers rely on the inductive detection of oscillating magnetic fields generated by precessing nuclear spins. The signal-to-noise ratio (SNR) is strongly dependent on the external field strength (*B*_0_), scaling proportional to $B_{0}^{7/4}$ (*1*). The spectral resolution also improves with increasing *B*_0_, since spectral splittings due to chemical shifts increase proportional to *B*_0_. This has motivated the development of increasingly large and expensive superconducting magnets to improve resolution and SNR, resulting in a twofold increase in field strength in the past 25 years (*2*). However, even for *B*_0_ ≳ 10 T, detection of microscale volumes often requires isotopic labeling, concentrated samples, and long experimental times (*2*).

To improve sensitivity for small volume samples, miniature inductive coils have been developed (*3*, *4*). This approach has enabled several advances including the spectroscopy of individual egg cells (*5*, *6*) and in vitro diagnostics based on NMR relaxometry (*7*). However, the present sensitivity and detection volumes are suboptimal for metabolic analysis of single mammalian cells (*8*) or incorporation into in-line microfluidic assays (*9*).

**Fig. 1:**
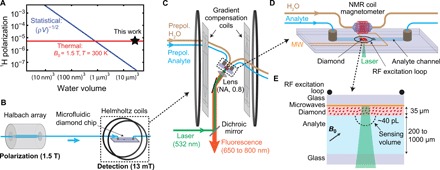
Microfluidic prepolarization NMR setup. (**A**) Comparison of statistical and thermal polarization of protons in water as a function of detection volume. The room temperature water proton density is ρ = 6.7 × 10^28^ m^−3^. (**B**) Prepolarization concept. Analyte is prepolarized by flowing it through a permanent magnet (1.5-T Halbach array). It is subsequently shuttled to a microfluidic chip housed in a stabilized, lower magnetic field (*B*_0_ = 13 mT, Helmholtz coils) where it is detected by NV NMR. (**C**) Detection setup. Prepolarized analyte flows to a microfluidic chip where it is stopped via fluidic switches (not shown), and the NV NMR signal is detected using a custom-built epifluorescence microscope with a numerical aperture (NA) of ∼0.8. A set of eight gradient compensation coils is used to eliminate first- and second-order magnetic field gradients along the field direction. The field is stabilized temporally using a coil-based NMR magnetometer in combination with low-inductance feedback coils wound around the main Helmholtz coils. (**D**) Microfluidic chip setup. The chip is constructed using glass and adhesives (section SV). Two fluidic lines pass to the detection region, one consisting of water (for NMR coil magnetometer) and the other with analyte (for NV NMR). A radio frequency (RF) excitation loop, placed in between the NMR coil magnetometer and the NV NMR sensor, excites nuclear spin coherence in both channels. The NMR coil magnetometer consists of a 3-mm-diameter coil wound around a ∼10-μl water volume. The RF excitation loop and NMR coil magnetometer were placed orthogonal to one another to minimize cross-talk. Copper microwave (MW) lines, printed on the interior of the glass chip, provide spin control over NV electron spins. (**E**) NV NMR geometry. An NV-doped diamond membrane (1 mm by 1 mm by 0.035 mm) is located on the surface of a microfluidic channel (width: 2 mm, height: between 0.2 mm and 1 mm) in contact with the analyte. Laser illumination (532 nm) bounces off the printed microwave line, and fluorescence (650 to 800 nm) is detected. The effective analyte detection volume is ∼40 pL (section SXII).

Quantum sensors based on nitrogen-vacancy (NV) centers in diamond have emerged as an alternative NMR detection modality because of their submicrometer spatial resolution and noninductive-based detection. Early implementations (*10*, *11*) used nanoscale fluctuations of nuclear magnetization (statistical polarization) to enhance polarization (Fig. 1A). However, nanoscale diffusion of the analyte across the sensing volume broadened the spectral distribution to ∼1 kHz, masking the informative spectral features arising from chemical shifts and *J*-couplings (*12*, *13*). The use of viscous solvents (*14*) improved the frequency resolution to ∼100 Hz, enabling the resolution of large chemical shifts at *B*_0_ = 3 T. While further improvements in resolution are possible by increasing the detection volume (*V*), these come at a steep cost in SNR since statistical polarization scales ∝*V*^−1/2^ (Fig. 1A).

Alternatively, at sufficiently large *V* and *B*_0_, the net thermal (Boltzmann) polarization becomes the dominant contributor to nuclear polarization (see Fig. 1A). Detection of thermal polarization was recently demonstrated (*15*) using an NV-based NMR spectrometer achieving a frequency resolution of ∼10 Hz operating at *B*_0_ = 88 mT. This resolution was sufficient to detect large spectral splittings due to proton chemical shifts and *J*-couplings, with a concentration sensitivity (defined throughout as the minimum detectable proton concentration for SNR = 3) of ∼370 M s^1/2^.

Here, we report an order-of-magnitude improvement in spectral resolution, 0.65 ± 0.05 Hz, and realize a concentration sensitivity of ∼27 M s^1/2^. This is accomplished by spatially separating the polarization and detection phases of the experiment in a microfluidic setup (*16*, *17*). Strong permanent magnets (1.5 T) are used to generate nuclear spin polarization. Detection is performed at 13 mT using Helmholtz coils, simplifying the task of stabilizing NMR linewidths to sub-hertz levels while enabling the use of high-sensitivity diamond quantum sensing protocols at low microwave frequencies (*15*). These improvements allow us to perform two-dimensional (2D) correlation spectroscopy (COSY) of liquid analytes within an effective ∼40-pL detection volume. The use of diamond quantum sensors as in-line microfluidic NMR detectors is a major step toward applications in mass-limited chemical analysis and single-cell biology. In combination with advances in dynamic nuclear polarization using external polarizing agents (*18*) and, potentially, optical hyperpolarization using NV centers (*19*–*24*), this platform may eventually enable NMR spectroscopy of metabolites at physiological concentrations with single-cell spatial resolution.

## RESULTS

Figure 1B shows the prepolarization concept. Fluid analytes are housed in a helium-pressurized container, which enables variable flow rates up to 50 μl/s. The analyte first flows through a 1.5-T Halbach array for a dwell time of ∼6 s. This time is longer than the longitudinal spin relaxation time of the analytes studied here [for water, *T*_1_ ≈ 3 s (*25*, *26*); see section SIX], leading to an equilibrium polarization of ∼5 × 10^−6^. The analyte then flows to a detection region where it is detected by NV NMR. For the analyte to retain the thermal polarization generated in the prepolarization step, the transfer must be performed adiabatically (the rate of change in the magnetic field angle should be much smaller than the nuclear spin angular frequency) and on a shorter timescale than *T*_1_ (*27*). Both conditions are satisfied in our experiment (section SVIII). Microfluidic switches ensure that the analyte is transferred to the detection region and then stopped for NMR detection (see section SVII).

Figure 1C depicts the detection setup. Helmholtz coils produce a magnetic field *B*_0_ = 12.935 mT, corresponding to a proton resonance frequency γ_p_*B*_0_ = 550.75 kHz, where γ_p_ = 42.577 MHz/T is the proton gyromagnetic ratio. A set of gradient compensation coils, consisting of eight separate current-carrying wire configurations, enables cancellation of first- and second-order magnetic field gradients along the field direction (see section SII). The magnetic field is temporally stabilized using a feedback loop incorporating a custom NMR coil magnetometer positioned just above the diamond detection volume (Fig. 1D). Prepolarized water continuously flows through a 3-mm-diameter NMR detection coil. The water’s proton nuclear precession is initialized by a π/2 pulse using the same radio frequency (RF) loop used for NV NMR. The inductively detected coil signal is amplified, digitized, and fit for the proton NMR frequency. The instantaneous magnetic field is inferred, and temporal deviations are actively compensated by altering the current in a pair of low-inductance compensation coils. With this system, we realize a temporal field stability of ∼1 parts per million (ppm) (∼0.6 Hz at the proton NMR frequency), limited by the accuracy of the NMR coil magnetometer (section SIV).

The microfluidic chip housing the diamond sensor is depicted in Fig. 1D. The components of the chip include a copper loop (printed on a glass slide) used to deliver microwaves, an RF excitation loop placed between the diamond and the feedback NMR coil, a microfluidic channel enclosing the diamond sensor and contacting analyte, and microfluidic ports to mate the external analyte tubing with the chip. An enlarged picture of the chip surrounding the diamond sensor is shown in Fig. 1E. A 20-μm-diameter laser beam excites NV centers throughout a 35-μm-thick diamond membrane. Finite-element magnetostatic modeling indicates that 50% of the NMR signal comes from a ∼40-pL hemispherical region of analyte above the optical axis (section SXII). By convention (*15*), we define this region as the effective detection volume. However, the total volume of analyte flowing through the entire apparatus is several milliliters, and the NV NMR detector repeatedly interrogates different ~40-pL portions of this much larger volume.

Several 35-μm-thick diamond membranes were used with 100 polished faces (1 mm by 1 mm). The membranes were formed from diamond chips grown by either high-pressure high-temperature synthesis or chemical vapor deposition and hosted an initial nitrogen density of 20 to 50 ppm. The chips were irradiated with 2-MeV electrons at a dose of ∼10^18^ cm^−2^ and subsequently annealed at 800 ° to 1100 ° C using the recipe described in (*12*). NV centers in the processed membranes exhibit a coherence time of 10 to 20 μs under an XY8-1 pulse sequence.

NV NMR detection was performed using a custom-built epifluorescence microscope (Fig. 1C). Linearly polarized pulses of laser light (0.3 W, 532 nm) polarize and detect the spin projection states of NV centers via their spin-dependent fluorescence. The fluorescence is spectrally filtered (650 to 800 nm) and imaged onto a photodetector, producing ∼10 μA of peak photocurrent. The diamond membranes are oriented so that one of the four possible NV axes is aligned with the magnetic field. The optically detected magnetic resonance (ODMR) transitions of these aligned NV centers is *D* ± γ_NV_*B*_0_, where *D* = 2.87 GHz is the NV zero-field splitting and γ_NV_ = 28.0 GHz/T is the NV gyromagnetic ratio. NV center spin states are manipulated using microwaves resonant with the lower-frequency transition of 2.51 GHz. Throughout, we set the microwave power to produce a π pulse length of 44 ns. The normalized peak-to-peak amplitude of the processed photodetector signal during Rabi oscillations is typically 8%. A half waveplate on the optical excitation path and a linear polarizer on the fluorescence path are adjusted to maximize the Rabi contrast (*28*).

**Fig. 2:**
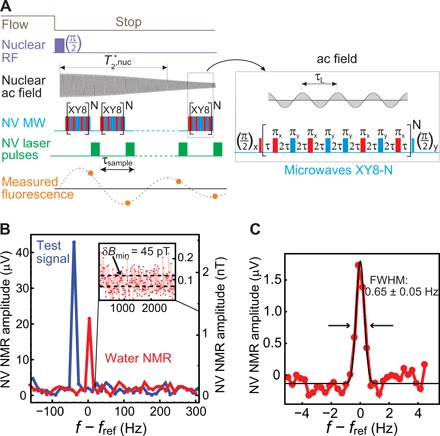
Characterization of prepolarized NV NMR. (**A**) The synchronized readout pulse sequence. It consists of a train of XY8-N pulses that perform successive phase measurements of the ac magnetic field produced by precessing nuclei. The measured fluorescence reflects an aliased version of the nuclear ac field projection. The entire sequence is repeated every 2.5 to 4.25 s (1.25 s for flow and the remainder for detection). (**B**) NV NMR spectra (absolute value of Fourier transform) of water (red) and an applied 2.5-nT amplitude test field (blue) for an effective acquisition time of 5.2 s (average of 60 traces; total measurement time, 150 s). The NMR signal amplitude obtained from the processed photodetector signal is recorded in μV. The conversion to magnetic field amplitude (in nT) is derived from the calibrated test field (see section SX). Inset: The SD of the noise floor reveals *aB*_min_ = 45 pT. From these data, we infer a minimum detectable concentration of 27 M s^1/2^ (SNR = 3). Incorporating all experimental dead time, the concentration sensitivity is ∼45 M s^1/2^ (section SXI). (**C**) A high-resolution NV NMR spectrum of water (imaginary part of Fourier transform) reveals a full width at half maximum (FWHM) linewidth of 0.65 ± 0.05 Hz. Data were obtained by averaging 60 traces, each 3 s long.

The pulse sequence used to detect NV NMR is depicted in Fig. 2A. It shares common traits with the synchronized readout scheme used in (**15*, *29*, *30**). A π/2 RF pulse (∼1 ms long), resonant with the proton spin transition, initializes nuclear spin precession, producing an exponentially decaying oscillating (ac) magnetic field with a nominal frequency *f*_ref_ = 1/τ*_L_* = 550.75 kHz. Subsequently, a series of XY8-5 microwave pulse sequences are applied to the NV centers to detect the nuclear ac field. Only the component of the nuclear ac field along the NV axis is detected (*15*). Each XY8-5 sequence contains 40 π pulses separated by τ*_L_*/2. After each XY8-5 sequence, a 3.4-μs laser pulse is applied to the NV centers for optical readout and repolarization. The first 0.5 μs of the readout fluorescence is used to measure the NV spin projection, and the final 1 μs is used for normalization to eliminate low-frequency intensity noise. Laser illumination is on for ∼10% of the total sequence. The average intensity (∼10 kW/cm^2^) was low enough to avoid damaging the microfluidic components and analyte.

Each NV readout nominally measures the initial phase of the nuclear ac field. A time series of the NV readouts yields an aliased version of the nuclear ac field projection with frequency *f*_alias_ = *f*_ref_ − *f*_sample_ × round(*f*_ref_/*f*_sample_), where *f*_sample_ = 1/τ_sample_ ≈ 24 kHz is the sampling frequency of NV readouts. Unlike the sequence used in (*15*), the duration of each XY8-5 sequence is held constant at the point of maximal sensitivity, while *f*_alias_ is varied up to the maximum frequency *f*_sample_/2 ≈ 12 kHz by adjusting a small dead time between readouts.

The sensitivity and spectral resolution limits of our apparatus were determined from measurements on deionized water. Figure 2B shows results of the sensitivity measurements. An ac magnetic field, with a calibrated amplitude of 2.5 nT (see section SX), was detuned slightly from *f*_ref_ and detected using the NV NMR pulse sequence. The resulting signal provides a calibrated conversion between field amplitude (in nT) and processed photodetector signal amplitude (in μV). The NV NMR signal from prepolarized water was then recorded under identical conditions. The Fourier transform of the water signal reveals an amplitude of 1.21 nT. Finite-element magnetostatic modeling predicts that a proton polarization of 4 × 10^−6^ would produce this signal strength (section SXII). This signifies that ∼80% of the maximum thermal polarization generated in the Halbach array (5.1 × 10^−6^ for 1.5 T at 300 K) is retained. The standard deviation (SD) of points near the resonance peak (inset of Fig. 2B) reveals a magnetic noise of 0.10 nT s^1/2^. This corresponds to a concentration sensitivity of 27 M s^1/2^ for SNR = 3. Between experiments, the concentration sensitivity varied by ∼50% depending on the fluorescence level, contrast, and NV coherence time of the diamond illumination region.

To optimize the spectral resolution, the gradient compensation coils (*31*) were adjusted until no perceptible decrease in NV NMR linewidth was observed. Figure 2C shows an NV-detected water NMR spectrum with one of the narrowest linewidths obtained. A Gaussian fit reveals a full width at half maximum of 0.65 ± 0.05 Hz. While this is a substantial improvement over previous studies, it is broader than the expected natural linewidth of water under our experimental conditions, ∼0.1 Hz (*32*). We attribute the discrepancy to residual temporal instability in *B*_0_ (section SIV).

**Fig. 3:**
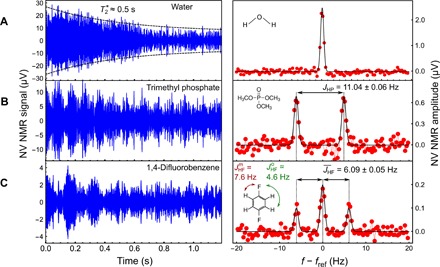
1D NMR. Time-domain (left) and frequency-domain (right) NV NMR signals for (**A**) water, (**B**) trimethyl phosphate (TMP), and (**C**) 1,4-difluorobenzene (DFB). Signals were averaged over ∼10^3^ traces for a total acquisition of ∼1 hour. A ∼1-kHz-bandwidth bandpass filter is applied to the time-domain data for better visualization. The frequency-domain spectra show the imaginary component of the Fourier transform. Each spectrum is fit with Gaussian functions (black lines). For TMP, we constrain the widths of both lines to be equal with a 1:1 amplitude ratio and find *J*_HP_ = 11.04 ± 0.06 Hz. For DFB, we constrain the widths of all three lines to be equal with a 1:2:1 amplitude ratio and find $\bar{J_{\text{HF}}}=6.09\pm 0.05\;\text{Hz}$.

To showcase the capabilities of our NV NMR spectrometer, we obtained proton NMR spectra of different fluid analytes. Figure 3A shows the time and frequency domain signals of water. The SNR is sufficient to resolve the decay in the envelope of the proton magnetization, from which we infer a spin dephasing time $T_{2}^{*}\approx 0.5\;s$, consistent with the sub-hertz linewidths observed in the frequency domain.

Figure 3B shows the NV NMR spectrum for trimethyl phosphate (TMP). The characteristic beats in the time domain and spectral splitting in the frequency domain are signatures of *J*-coupling. These splittings arise due to terms in the nuclear spin Hamiltonian of the form $J_{12}\vec{I_{1}}\cdot \vec{I_{2}}$, where ${\vec{I}}_{1}$ and ${\vec{I}}_{2}$ are the spin angular momenta of different nuclei. At *B*_0_ = 13 mT, couplings between spins of different isotopes (“heteronuclear” *J*-coupling) lead to well-defined splittings in the NMR spectra, whereas homonuclear *J* splittings are not resolved (*33*). The splitting (11.04 ± 0.06 Hz) in the TMP spectrum corresponds to the known heteronuclear *J*-coupling between the ^31^P nuclear spin and each of the equivalent ^1^H spins (*34*).

Figure 3C shows the NV NMR spectrum for 1,4-difluorobenzene (DFB). In DFB (inset), each proton is coupled to the nearest ^19^F atom with $J_{\text{HF}}^{m}=7.6\;\text{Hz}$ and the further ^19^F atom with $J_{\text{HF}}^{o}=4.6\;\text{Hz}$ (*35*). The spectrum exhibits an average of the two splittings, $\bar{J_{\text{HF}}}=6.09\pm 0.05\;\text{Hz}$, with a 1:2:1 amplitude ratio, consistent with previous reports (*35*, *36*).

Having established the ability to detect NMR spectra with sub-hertz resolution and high SNR, we next used our platform to perform 2D COSY NMR spectroscopy. Multidimensional NMR spectroscopy enables the determination of nuclear interactions within complex structures, even in cases where the corresponding 1D spectra are complicated or have ambiguous interpretation. It is widely used in applications ranging from metabolomics to protein structure identification (*37*, *38*).

**Fig. 4:**
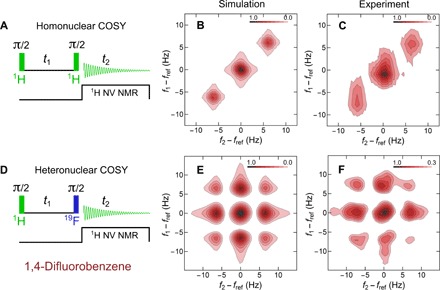
2D COSY NMR of DFB. (**A**) Homonuclear COSY pulse sequence, (**B**) simulated spectrum, and (**C**) experimental NV NMR spectrum of DFB. (**D**) A modified heteronuclear COSY sequence reveals off-diagonal peaks in both (**E**) simulation and (**F**) experiment. Color scales correspond to the normalized absolute value of the 2D Fourier transform. Vertical axes (*f*_1_ − *f*_ref_) correspond to the frequencies of the *t*_1_ dimension, and horizontal axes (*f*_2_ − *f*_ref_) correspond to the frequencies of the *t*_2_ dimension. In (C), 14 values of *t*_1_ in 0.021-s increments up to 0.294 s were used. Total acquisition time was 22 hours. In (F), 16 values of *t*_1_ in 0.021-s increments up to 0.336 s were used. Total acquisition time was 25 hours. In both cases, the *t*_2_ acquisition spanned from 0 to 1.25 s. All simulations were performed using the SPINACH package (*41*). Simulation and experimental data use the same windowing functions (see section SXV).

We performed two different variations of the 2D COSY experiment that probe the nuclear interactions within DFB. In the first case, homonuclear COSY (*39*, *40*) (shown in Fig. 4A), two π/2 pulses on the proton spins are separated by a variable evolution period, *t*_1_. Following the second pulse, the precessing proton magnetization is continuously recorded as a function of time, *t*_2_. The sequence is then iterated by incrementing *t*_1_ to build up a 2D array.

Figure 4 (B and C) shows the 2D Fourier transform of the resulting array for DFB alongside a simulated spectrum obtained by density matrix modeling (section SXV) using the SPINACH software package (*41*). Three diagonal peaks are observed, which are separated by $\bar{J_{\text{HF}}}=6.1\;\text{Hz}$. However, the absence of cross peaks indicates a lack of magnetization transfer between the spin states. This is expected since there is no difference in the chemical shift between the protons (*35*). A homonuclear COSY spectrum of TMP is presented in section SXIV.

In the second 2D NMR experiment on DFB, we used a modified heteronuclear COSY sequence where the second π/2 pulse is resonant with ^19^F nuclei (518.08 kHz) (Fig. 4D). As before, the pulses are separated by a variable evolution time, *t*_1_, and we tune our NV NMR sequence to selectively detect the proton precession as a function of *t*_2_. The simulated and experimental 2D Fourier transforms are shown in Fig. 4 (E and F). The presence of cross peaks separated by ∼6 Hz indicates that the ^19^F pulse mediates transfer of magnetization among the *J*-split proton spin states. The results are consistent with previous findings on DFB at Earth’s magnetic field (*36*). In section SXIII, we provide an analytical calculation of a two-spin model, which effectively describes these dynamics.

## DISCUSSION

The demonstration of sub-hertz resolution and multidimensional NMR paves the way for diamond quantum sensors to be used in applications such as in-line hyphenated analysis (*9*), single-cell metabolomics (*8*), and mass-limited pharmacodynamics (*38*). The high spatial resolution, epifluorescence imaging format of our sensor lends itself to parallelization, which could enable high-throughput chemical analysis or NMR imaging of cell cultures with single-cell resolution.

A limitation of the present sensor is that it would require substantial averaging times for detection of metabolites at physiological concentrations (micromolar to millimolar). In the short term, up to an order-of-magnitude improvement in NV NMR sensitivity may be realized by detecting at higher magnetic field (which would enable the use of longer, more sensitive XY8-N sequences) (*42*), improving the photon collection efficiency (*43*, *44*), and increasing the NV emission intensity and contrast through optimized diamond doping (*45*–*47*). Another order-of-magnitude improvement in concentration sensitivity is possible by using a superconducting magnet for prepolarization (*48*). The use of external polarizing agents may improve the sensitivity by up to two orders of magnitude (*18*), provided that these additives are compatible with the target assay. In the longer term, the largest gains in sensitivity may come from the use of optical hyperpolarization methods to transfer the near-unity NV electron spin polarization to the analyte noninvasively (*19*–*24*).

Another limitation is the use of a low external field, *B*_0_ = 13 mT, which restricts the ability to resolve spectral splittings due to chemical shifts. Chemical shift resolution could be improved using the present detection scheme by increasing *B*_0_ to ∼0.25 T (proton NMR frequency, ∼10 MHz). For higher fields, a different pulse sequence may be necessary, as the nuclear precession half-period becomes comparable to the achievable NV π pulse length (see Fig. 2A). In this regime, pulse sequences that sample nuclear precession via Ramsey interferometry (*14*), combined with sensitive NV detection of longitudinal nuclear magnetization (*10*), may be used.

Last, while our microfluidic NMR sensor has an effective detection volume of ∼40 pL, several milliliters of analyte are needed to fill the overall flow apparatus. Future microfluidic chips may miniaturize or omit the prepolarization step, use smaller microfluidic channels for detection, and/or use microdroplets to shuttle small samples within a larger fluidic system (*49*).

In summary, we demonstrated that diamond quantum sensors can be used in microfluidic NMR applications. We showed that separating polarization and detection steps enabled an order-of-magnitude improvement in spectral resolution (0.65 Hz) over previous diamond NMR studies, with a concentration sensitivity of ∼27 M s^1/2^. We used the platform to perform 2D NMR on fluid analytes and observed the transfer of magnetization mediated by heteronuclear *J*-coupling.

## MATERIALS AND METHODS

### NV NMR detection

A linearly polarized 532-nm green laser beam (Lighthouse Photonics Sprout-G 10 W) was used to excite NV centers. Laser pulses were generated by passing the continuous-wave laser beam through an acousto-optic modulator (CrystaLaser). A 0.8–numerical aperture aspheric lens was used to illuminate a 20-μm-diameter spot on the diamond and collect fluorescence. The fluorescence was separated from the excitation light by a dichroic mirror, passed through a linear polarizer, and focused by a 200-mm focal-length lens onto an amplified photodetector (Thorlabs PDB450A). Microwave pulses were generated using an I/Q modulated microwave generator (SRS SG384). RF nuclear π/2 pulses were generated by an arbitrary waveform generator (Teledyne LeCroy WaveStation 2012). A transistor-transistor logic (TTL) pulse card (SpinCore PBESR-PRO-500) was used to generate and synchronize the pulse sequence. Two data acquisition cards (National Instruments USB-3631) were used to digitize NV NMR and RF coil magnetometer signals. Helmholtz coils were driven by a HighFinesse Gmbh UCS 10/40 current source. A set of eight gradient compensation coils (NuevoMR LLC) was used to minimize spatial gradients in *B*_0_. The temporal drift of *B*_0_ was monitored with an NMR coil magnetometer (placed above the diamond detection volume) and stabilized using an additional pair of coils.

### Chip construction

Microfluidic chips were constructed from glass, epoxy, and double-sided tape. Copper traces were fabricated on a 1-mm-thick glass slide and connected via a nonmagnetic SubMiniature version A (SMA) solder jack to deliver microwaves. Two 0.5-mm-diameter holes were drilled into the slide to deliver fluid analytes to and from the chip. The diamond membranes (1 mm by 1 mm by 0.035 mm) were affixed to the slide (on the side containing copper traces) using epoxy and oriented such that one of the NV axes was aligned along *B*_0_ once positioned in the setup. The microfluidic channel was defined by a spacer layer constructed in one of two ways. In the first method (used for DFB and TMP), a second 1-mm-thick microscope slide served as the walls of the microfluidic channel. The slide was cut to produce a ∼35-mm-long channel spanning the inlet and outlet holes. A slight taper was introduced at each end, with the widest part of the channel (∼2 mm) in the center where the diamond was positioned. In the second method (used for water), a channel with similar length and width was cut from double-sided tapes (UltraTape 1510). The latter method enabled construction of thinner channels (0.2 to 1 mm thick). The spacer layer was glued (for the glass spacer) or adhered (tape spacer) onto the copper-trace slide such that the diamond was positioned in the channel. A 0.1- to 0.2-mm-thick coverslip was glued or adhered to the top of the spacer layer to seal the channel. A helium-pressurized container controlled the flow of analyte. Polyetheretherketone (PEEK) tubing was used to deliver analyte through the prepolarization setup, and rubber stoppers were used to mate the tubing with the inlet and outlet holes of the chip.

### Sample preparation

Both TMP (99%) and DFB (99%) were purchased from Sigma-Aldrich. Water samples were deionized, and all analytes were degassed in a sonicator before measuring. Additional details on the experimental setup, sample handling, construction methods, and pulse timing can be found in the Supplementary Materials.

## Supplementary Material

http://advances.sciencemag.org/cgi/content/full/5/7/eaaw7895/DC1

Download PDF

## SUPPLEMENTARY MATERIALS

Supplementary material for this article is available at http://advances.sciencemag.org/cgi/content/full/5/7/eaaw7895/DC1 Section SI. NV NMR detection apparatus Section SII. Magnetic field gradient compensation Section SIII. Gradients due to magnetic susceptibility mismatch of sensor components Section SIV. NMR coil magnetometer feedback system Section SV. Microfluidic chip fabrication Section SVI. Sample preparation Section SVII. Microfluidic flow and switch timing Section SVIII. Adiabaticity considerations Section SIX. Optimization of flow rates Section SX. Magnetic field calibration Section SXI. Concentration sensitivity Section SXII. NMR field amplitudes and effective sensing volume Section SXIII. Analytical calculation for heteronuclear COSY Section SXIV. 2D homonuclear COSY of TMP Section SXV. SPINACH simulations and windowing functions for 2D NMR Fig. S1. Magnetostatic modeling of a diamond immersed in water. Fig. S2. Histogram of fitted central frequencies obtained from the NMR coil magnetometer for a typical measurement. Fig. S3. NMR signal strength dependence on flow rate and RF pulse length. Fig. S4. Saturation curve of the NV NMR. Fig. S5. NV NMR spectrum of water. Fig. S6. Nuclear ac magnetic field projection amplitude (integrated across the sensor volume) as a function of water volume. Fig. S7. Experimental homonuclear COSY spectrum of TMP. Table S1. Values of the different *J*-couplings in a DFB molecule used in the simulation.
