# Supplementary material for: Two-dimensional nuclear magnetic resonance spectroscopy with a microfluidic diamond quantum sensor
Source: Sci Adv. 2019 Jul 26;5(7):eaaw7895. doi: 10.1126/sciadv.aaw7895 (PMC6660203; doi:10.1126/sciadv.aaw7895)
Supplement: http://advances.sciencemag.org/cgi/content/full/5/7/eaaw7895/DC1 [file supp_5_7_eaaw7895__index.html]

Science Advances | Science AdvancesAAASSearchScience AdvancesMenu

## Supplementary Materials

**This PDF file includes:**

- Section SI. NV NMR detection apparatus
- Section SII. Magnetic field gradient compensation
- Section SIII. Gradients due to magnetic susceptibility mismatch of sensor components
- Section SIV. NMR coil magnetometer feedback system
- Section SV. Microfluidic chip fabrication
- Section SVI. Sample preparation
- Section SVII. Microfluidic flow and switch timing
- Section SVIII. Adiabaticity considerations
- Section SIX. Optimization of flow rates
- Section SX. Magnetic field calibration
- Section SXI. Concentration sensitivity
- Section SXII. NMR field amplitudes and effective sensing volume
- Section SXIII. Analytical calculation for heteronuclear COSY
- Section SXIV. 2D homonuclear COSY of TMP
- Section SXV. SPINACH simulations and windowing functions for 2D NMR
- Fig. S1. Magnetostatic modeling of a diamond immersed in water.
- Fig. S2. Histogram of fitted central frequencies obtained from the NMR coil magnetometer for a typical measurement.
- Fig. S3. NMR signal strength dependence on flow rate and RF pulse length.
- Fig. S4. Saturation curve of the NV NMR.
- Fig. S5. NV NMR spectrum of water.
- Fig. S6. Nuclear ac magnetic field projection amplitude (integrated across the sensor volume) as a function of water volume.
- Fig. S7. Experimental homonuclear COSY spectrum of TMP.
- Table S1. Values of the different *J*-couplings in a DFB molecule used in the simulation.

Download PDF

**Files in this Data Supplement:**

- Adobe PDF - aaw7895\_SM.pdf
