# Supplementary material for: Two-dimensional nuclear magnetic resonance spectroscopy with a microfluidic diamond quantum sensor
Source: Sci Adv. 2019 Jul 26;5(7):eaaw7895. doi: 10.1126/sciadv.aaw7895 (PMC6660203; doi:10.1126/sciadv.aaw7895)
Supplement: Download PDF [file aaw7895_SM.pdf]

Science Advances
AAAS

advances.sciencemag.org/cgi/content/full/5/7/eaaw7895/DC1

Supplementary Materials for

Two-dimensional nuclear magnetic resonance spectroscopy with a microfluidic diamond quantum sensor

Janis Smits, Joshua T. Damron, Pauli Kehayias, Andrew F. McDowell, Nazanin Mosavian, Ilja Fescenko, Nathaniel Ristoff, Abdelghani Laraoui, Andrey Jarmola*, Victor M. Acosta*

*Corresponding author. Email: andrey.jarmola@odmrtechnologies.com (A.J.); vmacosta@unm.edu (V.M.A.)

Published 26 July 2019, *Sci. Adv.* **5**, eaaw7895 (2019)
DOI: 10.1126/sciadv.aaw7895

This PDF file includes:

Section SI. NV NMR detection apparatus
Section SII. Magnetic field gradient compensation
Section SIII. Gradients due to magnetic susceptibility mismatch of sensor components
Section SIV. NMR coil magnetometer feedback system
Section SV. Microfluidic chip fabrication
Section SVI. Sample preparation
Section SVII. Microdluidic flow and switch timing
Section SVIII. Adiabaticity considerations
Section SIX. Optimization of flow rates
Section SX. Magnetic field calibration
Section SXI. Concentration sensitivity
Section SXII. NMR field amplitudes and effective sensing volume
Section SXIII. Analytical calculation for heteronuclear COSY
Section SXIV. 2D homonuclear COSY of TMP
Section SXV. SPINACH simulations and windowing functions for 2D NMR
Fig. S1. Magnetostatic modeling of a diamond immersed in water.
Fig. S2. Histogram of fitted central frequencies obtained from the NMR coil magnetometer for a typical measurement.
Fig. S3. NMR signal strength dependence on flow rate and RF pulse length.
Fig. S4. Saturation curve of the NV NMR.
Fig. S5. NV NMR spectrum of water.
Fig. S6. Nuclear ac magnetic field projection amplitude (integrated across the sensor volume) as a function of water volume.
Fig. S7. Experimental homonuclear COSY spectrum of TMP.
Table S1. Values of the different *J*-couplings in a DFB molecule used in the simulation.

Section SI. NV NMR detection apparatus

A 532 nm green laser (Lighthouse Photonics Sprout G-10W) beam was used to excite NV centers. An aspheric lens (Thorlabs ACL12708U, NA$\approx$ 0.8) focused the 0.3 W laser beam to illuminate a 20 µm diameter patch of diamond. Red fluorescence was separated from the excitation light by a dichroic mirror, passed through a linear polarizer (to improve fluorescence-detected spin contrast), and detected by an amplified photodetector (Thorlabs PDB450A) with 4 MHz bandwidth. A HighFinesse Gmbh UCS 10/40 ultra-low noise current source was used to drive the Helmholtz coils with 9.4 A.

The experiment was controlled by a TTL pulse card (PBESR-PRO-500 by SpinCore). Laser pulses were generated by passing the continuous-wave laser beam through an acousto-optic modulator (AOM, CrystaLaser). The modulator was driven by a 100 MHz RF source (Trinity Power TPI-1001-B). The source's amplitude was modulated on a $\lesssim$ 10 ns timescale using a switch (Mini-Circuits ZASWA-2-50DR). It was subsequently amplified to 1 W and delivered to the AOM.

Microwave pulses were generated using an I/Q modulated microwave generator (SRS SG384). The microwave amplitude and phase were controlled on a $\lesssim$ 10 ns timescale using a series of TTL controlled switches. The NV π pulse length was set to 44 ns and $\pi/2$ pulse length was 22 ns. The microwaves subsequently passed through an amplifier (Mini-Circuits ZHL-16W-43+) and circulator and were connected to the NV NMR chip.

RF pulses used for NMR $\pi/2$ pulses were generated by an arbitrary waveform generator (AWG, Teledyne LeCroy WaveStation 2012). The pulse timing was controlled by a trigger pulse from the TTL pulse card. A pulse signal from the AWG was also used for synchronizing the timing of the microfluidic flow switches. NMR $\pi/2$ pulses were typically $\sim$1 ms long and applied on nuclear spin resonance (550750 Hz for ^{1}H and 518082 Hz for ^{19}F).

Two data aquisition (DAQ) cards (NI USB-6361) were used. One DAQ was used for NV NMR data acquisition, and the other was used for the NMR coil magnetometer and temporal feedback. The clocks of the two DAQs and TTL pulse card were synchronized using low-drift oven-controlled crystal oscillators. For NV NMR, the photodetector signal was digitized and sychronized to the overall pulse sequence by using the TTL pulse card to generate the sample clock for the NV NMR DAQ.

Section SII. Magnetic field gradient compensation

The magnetic field was stabilized spatially using a set of eight gradient compensation coils (built by NuevoMR, LLC.), driven by two 4-channel power supplies (Instek GPD-4303S) producing currents in the 0–0.5 A range. The compensation coils were constructed according to the design described in Ref. [17]. Three channels compensated the linear field gradients, and the other 5 channels compensated second-order gradients. The design was modified slightly by replacing the $X^2 - Z^2$ and $Y^2 - Z^2$ coils with Z^2 $(= 3Z^2 - X^2 - Y^2)$ and $X^2 - Y^2$. Here the coils are labeled according to the Cartesian representation of the spherical harmonic terms in the field expansion, Ref. [17]. The spacing between the shim planes was 80 mm. Five turns of 24 AWG enameled magnet wire were laid out into double sided adhesive tape attached to 0.005" thick FR4 fiberglass boards, following a template visible through the FR4. Multiple coils were laid out and then sealed under epoxy, with a separate FR4 layer on top. The coils produce correction fields of 1.2–1.6 µT/mm/A for the linear terms, and $0.05 - 0.1$ µT/mm^2/A for the second-order terms.

Section SIII. Gradients due to magnetic susceptibility mismatch of sensor components

To estimate the contribution of susceptibility mismatches between the sensor and analyte, we performed finite-element magnetostatic simulations on a simplified two-dimensional model of the sensor, Fig. S1(a). A 35×150 µm^2 diamond membrane, with volume susceptibility $\chi_V \approx 2.2\times10^{-5}$, was placed in the center of a 1×1 mm^2 volume of water (volume susceptibility $\chi_V \approx 9.6 \times 10^{-6}$). Constant inward flux density boundary conditions were imposed on the boundaries at $y = \pm 500$µm and zero flux conditions were imposed on the remaining two boundaries. The relative change in magnetic field of the entire modeled region is shown in Fig. S1(a). Line cuts of the relative field through the analyte detection region are shown in Fig. S1(b,c).

While the fringe field near the edges of the diamond produce a relative change in field of a few parts per million, near the center of the diamond, the field variation is only at the ppb level. Since our analyte detection region is near the center of a much larger diamond chip, we therefore do not expect susceptibility mismatch to play a significant role in the NMR line broadening.

Section SIV. NMR coil magnetometer feedback system

For temporal feedback, the NMR coil magnetometer was tuned to proton NMR resonance using an LC circuit. The coil resonator had a quality factor of $\sim$ 20 and an impedance of $\sim$10 kHz. The output passed through a low-noise current amplifier (SRS 560) and was digitized by the feedback DAQ.

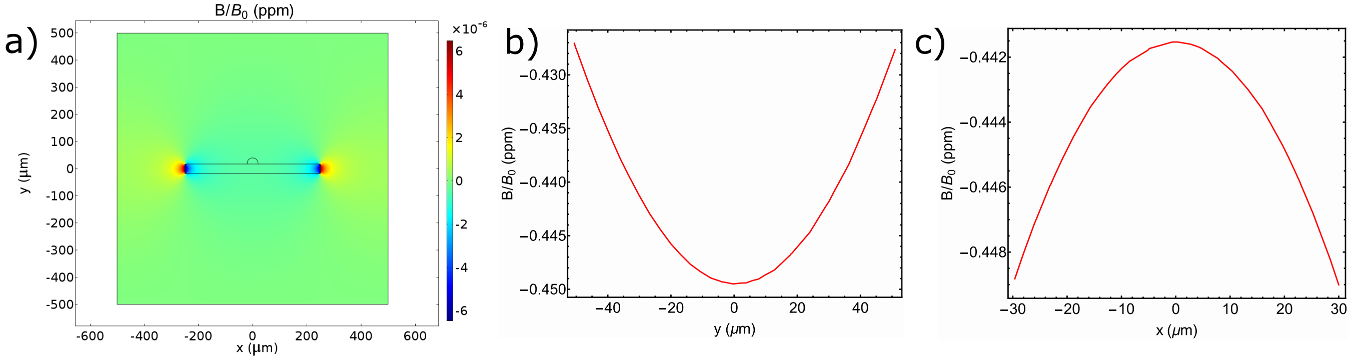

Fig. S1. Magnetostatic modeling of a diamond immersed in water. a) 2D map of the relative magnetic field around a diamond immersed in water. b) Line cut of the relative magnetic field at $x = 0$ c) Line cut of the relative magnetic field for the value of y located 10 μm above the diamond.

After each experiment with NV detection, a proton $\pi/\ 2$ pulse was applied, and the NMR coil magnetometer signal was digitized and converted to a power spectrum. The water NMR central frequency was calculated by fitting the power spectrum. The field was actively stabilized temporally by delivering 0–0.5 A of current to a pair of coils wound around the main Helmholtz coils. This current was delivered by a Thorlabs LDC220C current source, with its instantaneous current output controlled via an external analog modulation input. After each NMR coil magnetometer acquisition, the current in the feedback coils was adjusted to minimize detuning from the target central frequency.

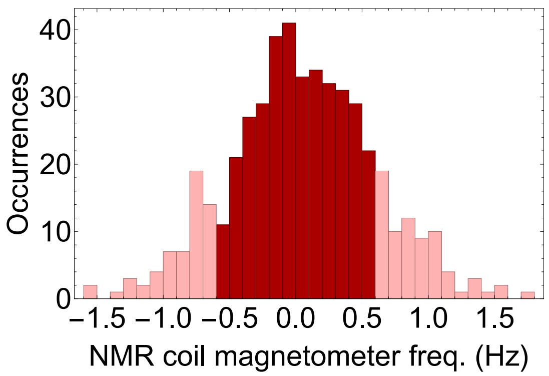

Fig. S2. Histogram of fitted central frequencies obtained from the NMR coil magnetometer for a typical measurement. Whenever the observed deviation is larger than a threshold value ($\pm$0 6 Hz in this case) the corresponding NV NMR measurement is discarded.

During 1D and 2D NV NMR measurements, the deviation of the feedback value from the desired value was monitored and whenever it was larger than a threshold value the corresponding NV measurement would be discarded. We typically set the threshold so that $\lesssim 25\%$ of total data was discarded. A histogram of NMR magnetometer frequency values obtained over the course of a few hours is shown in Fig. S2. The spread of deviations in field values is consistent with the 0.5–1 Hz linewidths observed in NV NMR experiments. The spread in values is due to a combination of (i) uncertainty in the fitted NMR magnetometer peak positions ($\sim \pm 0.3$ Hz) and (ii) real changes in field that occur on a faster timescale than the feedback bandwidth ($\sim$0.3 Hz). Regarding (i), although the RF excitation loop and NMR coil magnetometer were placed orthogonal to one another to minimize crosstalk, noise from residual crosstalk could still be a limiting factor in the NMR coil magnetometer's sensitivity.

The diamond NMR detection and NMR coil magnetometer phases of the experiment were separated temporally. The NMR coil magnetometer was only operated during the 1.25 second dead time when freshly polarized analyte was being shuttled into the diamond detection region. Thus the NMR coil magnetometer had no effect on the diamond NMR signal and vice versa.

Section SV. Microfluidic chip fabrication

The top surface of the microfluidic chips used in this study consisted of a 1-mm-thick microscope slide. Copper loops were fabricated on the slide and connected via a non-magnetic SMA solder jack to deliver microwaves. Two holes (an inlet and outlet), 0.5 mm in diameter, were then drilled into the slide, using a diamond-tipped drill bit. These inlet/outlet holes were used to deliver fluid analytes to and from the chip. The diamond was then glued on top of the copper loops and oriented such that one of the NV axes would be aligned along the Helmholtz magnetic field once positioned in the setup.

The microfluidic channel was defined by a spacer layer constructed in one of two ways. In the first method, a second 1-mm-thick microscope slide served as the walls of the microfluidic channel. The slide was cut to produce a $\sim$35 mm long channel spanning the inlet hole, diamond, and outlet hole. A slight taper was introduced at each end, with the widest part of the channel ($\sim$2 mm) in the center where the diamond was positioned. In the second method, a channel with similar length and width was cut from several layers of double-sided tapes (UltraTape 1510). The latter method enabled construction of thinner channels (0.2–1 mm thick).

The spacer layer was then either glued (in the case of the glass slide spacer) or adhered onto the copper coated slide with the affixed diamond. A 0.1–0.2 mm thick coverslip was glued or adhered to the top of the spacer layer to seal the channel. The choice of two channel fabrication strategies arose from practical reasons associated with the analytes. Both TMP and DFB diffuse through and dissolve the plastic double-sided tapes. This can be partially mitigated by coating the channel with epoxy, a strategy we employed for experiments on TMP. However the chemical resistance was insufficient for long measurements on DFB, so we used the glass spacer design for experiments with DFB. A two-component Gorilla Epoxy was used for all gluing steps.

Holes were drilled into two rubber stoppers to accommodate the 1/16 inch outer diameter PEEK tubing used for analytes. The stoppers were then attached with double sided tape (cutting a small hole in the tape for the tube opening) to the copper coated slide ensuring the tubing was aligned with the inlet/outlet holes. To reinforce the position and ensure a good seal, pressure was then applied to the stoppers using a piece of PCB board screwed into the Aluminum housing built for the entire chip.

On the inlet stopper, a second hole orthogonal to (and slightly offset from) the analyte PEEK tubing hole was drilled. The prepolarized water sample used for the feedback NMR coil magnetometer was fixed to this hole in such a way that the magnetometer detection volume was displaced $\sim$3 mm from the diamond NV NMR detection region. Finally, a single-turn RF excitation loop was glued to the inlet stopper such that it lied between the diamond and NMR coil magnetometer. This loop was used to simultaneously excite the analyte in the microfluidic chip and the water running through the NMR coil magnetometer.

Section SVI. Sample preparation

Both trimethyl phosphate (99%) and 1,4-difluorobenze (99%) were purchased from Sigma Aldrich. All water samples were deionized before measuring. All analytes were degassed in a sonicator for a minimum of 0.5 hours before measuring. Water was typically degassed for roughly 2 hours at an elevated temperatures (40–50° C), while trimethyl phosphate and difluorobenzene were kept at room temperature.

Section SVII. Microdluidic flow and switch timing

After sonication, the analyte was placed in a 600 mL glass container with a screwtop lid that was pressurized under helium to drive flow. The container was pressurized anywhere from 60 to 100 PSI to achieve the desired flow rates, which varied from analyte to analyte. PEEK tubing carried analytes from the pressurized container to a Fluigent two-way switch placed before the prepolarizing Halbach array. Following the switch, tubing went into the Halbach array and was wound in several loops before exiting. The total volume of fluid in the Halbach was $\sim$0.1 mL. From the Halbach, $\sim$35 μL of tubing ran to the microfluidic chip housing the diamond. Additional PEEK tubing was connected from the outlet of the chip, passed through a second fluidic switch (synchronized with the first switch) and exited into an exhaust container.

For the NMR coil magnetometer, a similar microfluidic path was built with the exception that no switch was installed for stop-flow. Water flowed continuously through this line throughout all experiments.

The timing of the fluidic switches was synchronized with the RF pulses and NV detection scheme. First, the switches were turned to the flow position for 1.25 s to replenish the NV NMR detection region with freshly polarized analyte. The switches were then turned off, followed by a variable delay to allow for settling of the fluid at the diamond interface. Thereafter, the RF pulse sequence was applied, followed by a minimum detection period of 1.25 s for NMR detection. At the end of the NMR detection sequence, a final RF pulse was applied for the feedback NMR coil magnetometer (NV detection is OFF at this stage), and the switches were activated for reflow. The entire sequence was repeated in a loop to enable signal averaging.

Section SVIII. Adiabaticity considerations

For optimum polarization retention, it is important to ensure that the spins remain aligned with the external magnetic field while transitioning from the prepolarization region to the detection region. This adiabaticity condition holds if the rate of change in the magnetic field direction is much smaller than the nuclear spin angular frequency [12]. To estimate if this condition holds for our experiments, we compare the proton spin angular frequency at the smallest field experienced during transit (~0.3 mT, corresponding to a spin angular frequency of $\sim 8\times 10^4$ rad/s) to the rate of change of field angle experienced by the fastest moving spins in the microfluidic system.

The narrowest tubing (corresponding to fastest flow) used in this experiment is $R = 90$ µm radius PEEK tubing and the fastest volumetric flowrates used is $Q = 50$ µL/s = 50 mm^3/s. Assuming laminar flow, the peak velocity at the center of the tube is:

$$v_{max} = \frac{2Q}{\pi R^2} \approx 4 \times 10^3 \text{ mm/s}. \tag{S1}$$

The most abrupt change in field angle occurs in our setup immediately after the Halbach array, where the field angle changes by $\sim\pi/2$ radians over ~40 mm. The fluid thus experiences a rate of change in field angle of approximately $\pi/2$ rad. $\frac{4\times 10^3 \text{ mm/s}}{40 \text{ mm}} = 150$ rad./s. This angular rate is more than two orders of magnitude smaller than the spin precession angular rate, meaning the spins follow the field adiabatically.

Section SIX. Optimization of flow rates

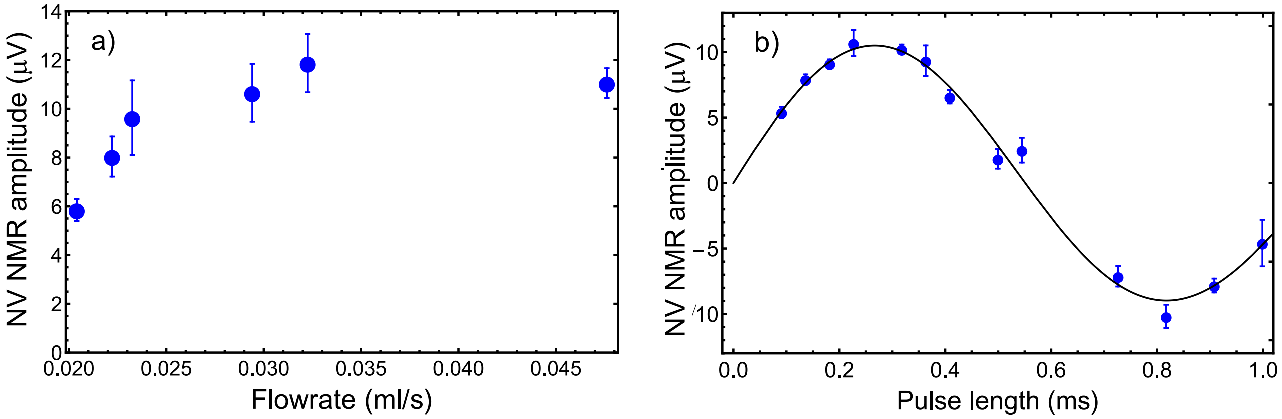

Fig. S3. NMR signal strength dependence on flow rate and RF pulse length. a) Dependence of water NV NMR amplitude on volumetric flow rate. The experiment was run by recording NV NMR signals as a function of flow rate (varied by adjusting the pressure of the analyte reservoir). The reflow time between NV NMR sequences was set to 3.5 s. b) Water NV NMR amplitude as a function of applied RF pulse length. The sinusodial nature of the curve indicates that the water proton spins are coherently driven by the RF pulses.

The analyte flow rate was optimized to efficiently transfer nuclear spin polarization from the Halbach array to the NV detection region. The ideal flow conditions ensure that there is sufficient "residence" time in the Halbach array, to fully polarize the sample, followed by fast delivery to the detection region before significant T_1 relaxation occurs. We measured the water NV NMR amplitude as a function of the flow-rate to find the optimal values, Fig. S3(a). Flow rates in the 25–50 µL/s range led to optimal polarization transfer. Flow rates were kept in this range for the reported experiments.

The data in Fig. S3 (a) can also be used to make a rough estimate of T_1 of the de-ionized water used in our experiments. To make this estimate, we assume that the water is fully polarized after the Halbach array and the subsequent flow velocity is constant across the lateral profile of the channels. The NV NMR amplitude should then be $\propto 1 - e^{-V_{\rm Hd}/(QT_1)}$, where $V_{\rm Hd} \approx 45$ µL is the volume of water between the Halbach array and detection region and Q is the volumetric flowrate. Since the NMR amplitude is reduced by ~50% when the flowrate is $Q = 20$ µL/s, this implies $T_1 \approx 3$ s. This estimate is consistent with T_1 values reported in bulk NMR under similar conditions [41, 46].

To calibrate the nuclear $\pi/2$ pulse length, we measured the water NV NMR amplitude as a function of the applied RF pulse length, Fig. S3(b). The well-behaved sinusodial dependence is a sign that analyte was fully stopped within the excitation loop volume before detection. In continuous flow (data not shown) we did not see this behavior; instead a slowly building signal with a very slow decay was observed indicating that new sample was entering the excitation and detection volumes on the timescale of the experiment. No clear $\pi/2$ pulse length could be inferred. Thus using

stop-flow was particularly important for the 2D NMR experiments, where well-defined nuclear pulse sequences are required.

Section SX. Magnetic field calibration

In Fig. 2 (b) of the main text, an ac test magnetic field was applied to calibrate the NV NMR response and corresponding voltage reading from the processed photodetector signal. The amplitude of this test field was calibrated using two complementary methods. In the first method, a DC current was applied to the test loop and the shift of the NV ODMR peaks was used to infer the conversion between current and the projection of the ac field amplitude along the NV axis, B_{ac}. In the second procedure, B_{ac} was calibrated by determining the ac current amplitude needed to maximize the oscillation amplitude of the NV NMR signal, i.e. where the phase accumulation during a single XY8-5 sequence is equal to $\pi/2$.

The time-domain NV NMR fluorescence signal depends on the ac magnetic field amplitude, B_{ac} as:

$$F(t) = F_0[1 + C\sin(4B_{ac}\gamma_{NV}\tau_{tot})\cos(2\pi(f - f_{alias})t + \phi_0)], \tag{S2}$$

where F_0 is the mean fluorescence intensity, $C \approx 0.01$ is the maximum fluorescence contrast of the XY8-5 sequence, f is the frequency of the ac magnetic field, f_{alias} is the aliasing frequency of the readout sequence, $\tau_{tot} = 24.16$ µs is the total phase accumulation time during a single XY8-5 sequence, and ϕ_0 is the phase of the applied oscillating magnetic field at the start of the acquisition sequence. We triggered the test field so that ϕ_0 is the same for each experiment, as is the case for detection of the nuclear ac field.
Evidently the maximum oscillation amplitude of $F(t)$ occurs for values of B_{ac} that satisfy $\sin(4B_{ac}\gamma_{NV}\tau_{tot}) = 1$.
This occurs when:

$$B_{ac,max} = \pi/(8\gamma_{NV}\tau_{tot}). \tag{S3}$$

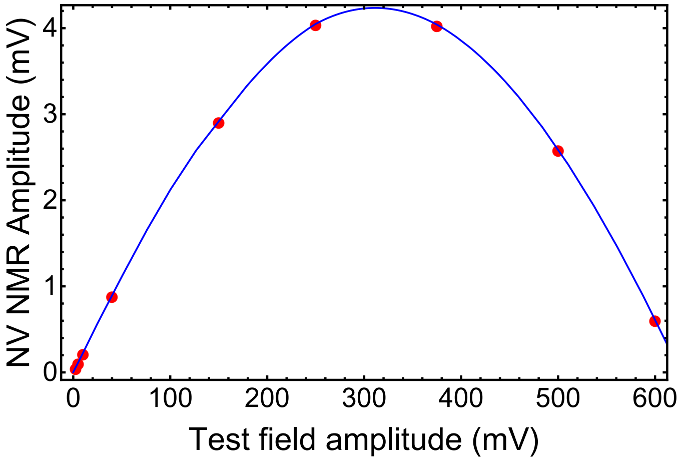

Fig. S4. Saturation curve of the NV NMR.

To determine the conversion of test field current amplitude and magnetic field, we acquired NV NMR sequences for different values of the voltage applied to the test loop. We determined the fluorescence oscillation signal amplitude by fitting the Fourier transform of the NV signal. Fig. S4 shows a plot of the NV signal amplitude as a function of test field amplitude. From this measurement we were able to calibrate the magnetic field value of the applied test signal used in Fig. 2(b) of the main text.

Section SXI. Concentration sensitivity

In Fig. 2 (b) of the main text, the data were acquired for 125 s while the experiment was repeated every 2.5 s for a total of 60 scans. To calculate the concentration sensitivity, we used only the first 0.087 s of NV NMR data, so the effective acquisition times was 60×0.085 s = 5.2 s. The final concentration sensitivity of 27 M $s^{1/2}$ was calculated using only this effective acquisition time, while neglecting dead time from the remainder of the pulse sequence and flow time. This estimate of concentration sensitivity is valid if one assumes that the transit time between prepolarization and detection phases can be neglected and the fluid can be flowed and stopped instantaneously. We anticipate that the transit times could be several orders of magnitude faster than we used here by optimizing the experimental geometry.

Fluidic switches with 20 ms rise/fall time are commercially available, so this estimate is within reach for an optimized experiment.

To calculate the more conservative concentration sensitivity, incorporating all experimental dead times, we use the full NV NMR signal and apply a windowing function to optimize SNR. Specifically, we apply a Lorentz-to-Gauss windowing function of the form:

$$W(t) = e^{\alpha t} e^{-(\beta t)^2}, \quad \alpha \geq 0, \beta \geq 0, \tag{S4}$$

to the time domain data and compute the Fourier Transform, Fig. S5. The signal is then defined as the discrete sum of the Fourier signal in a $[-7\text{ Hz}, +7\text{ Hz}]$ interval around the central maximum. The noise is defined as the standard deviation of the signal-free part of the spectrum multiplied by the square root of the number of points in the signal window. Using this method we find a SNR of 88 for the full 60×2.5 s = 150 s duration of the experiment. This corresponds to a SNR of 7.2 for 1 second integration or a concentration sensitivity of 45 M $s^{1/2}$. This conservative estimate is less than a factor of two worse than the optimal estimate (27 M $s^{1/2}$).

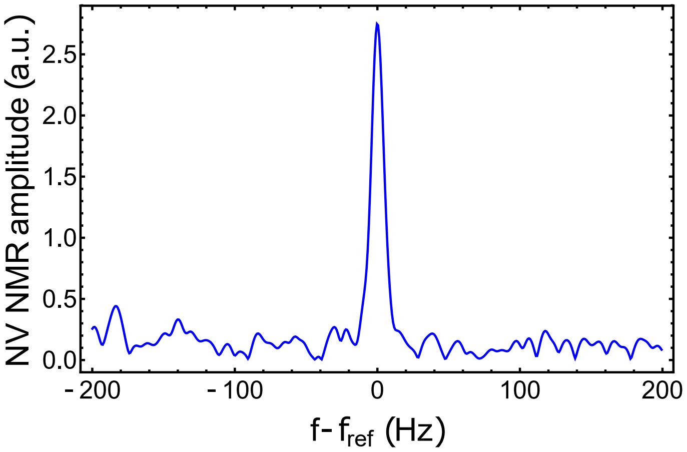

Fig. S5. NV NMR spectrum of water. A Lorentz-to-Gauss windowing function is applied with $\alpha = 16$ s^{-1} and $\beta = 13.5$ s^{-1}. The spectrum represents an average of 60 scans, with a scan repetition period of 2.5 s, corresponding to a total experimental acquisition of 150 s. The computed SNR is 88, corresponding to a concentration sensitivity of 45 M $s^{1/2}$.

Section SXII. NMR field amplitudes and effective sensing volume

A finite-element magnetostatic model was developed to estimate the nuclear ac field amplitude and effective detection volume. The illuminated NV sensor volume was modeled as a cylinder with a 20 μm diameter and 35 μm height defined by the thickness of the diamond and the laser beam area. The water volume was modeled in one of two ways. In the first case, we approximated it as a hemisphere with a varying radius. While this is a reasonable model for picoliter volumes, it neglects channel geometry and the field homogeneity of the nuclear RF excitation coil. Knowledge of these factors is needed to model the total effective sample volume. For this purpose, we calculated the transverse magnetic field produced by the RF excitation coil. We modeled the total effective sample volume as the volume where a nuclear RF excitation pulse produces a rotation between $\pi/3$ and $2\pi/3$ for the water nuclei. These bounds were selected so that the transverse component of the excited nuclear magnetization has an amplitude at least 50% of that produced by a true $\pi/2$ pulse. Under these assumptions, the total effective sample volume resembles a cylinder with radius and height of 1 mm. Those dimensions are comparable to or smaller than the true channel dimensions used for most of the manuscript, so there is no need to constrain the volume further. We note that this method of calculating total effective sample volume is still an approximation, as it neglects the effects of differential polarization across the channel (due to the Laminar flow velocity profile) and uncompensated spatial gradients in B_0. Curie's law was used to estimate the transverse nuclear magnetization following a $\pi/2$ pulse:

$$M = \frac{\rho \mu^2 B_H}{k_B T}, \tag{S5}$$

where $\rho = 6.7{\times}10^{28}$ m^{-3} is the proton spin density, μ is the proton magnetic moment, k_B is the Boltzmann constant, $T = 300$ K is the temperature, and $B_H = 1.5$ T is the polarizing magnetic field. The model outputs the component of the nuclear ac magnetic field amplitude along the NV axis, integrated over the NV sensor volume.

The nuclear ac field projection amplitude (integrated over the NV sensor volume) as a function of water volume is shown in Fig. S6(a). Evidently, the total effective sample volume (cylinder with 1 mm radius and 1 mm height) produces a similar signal strength to a hemispherical sample of comparable volume. We define the effective detection volume as the hemispherical volume of water that generates a nuclear ac field projection amplitude equal to half of that produced by the total effective sample volume. By this definition, the effective detection volume is ∼40 pL.

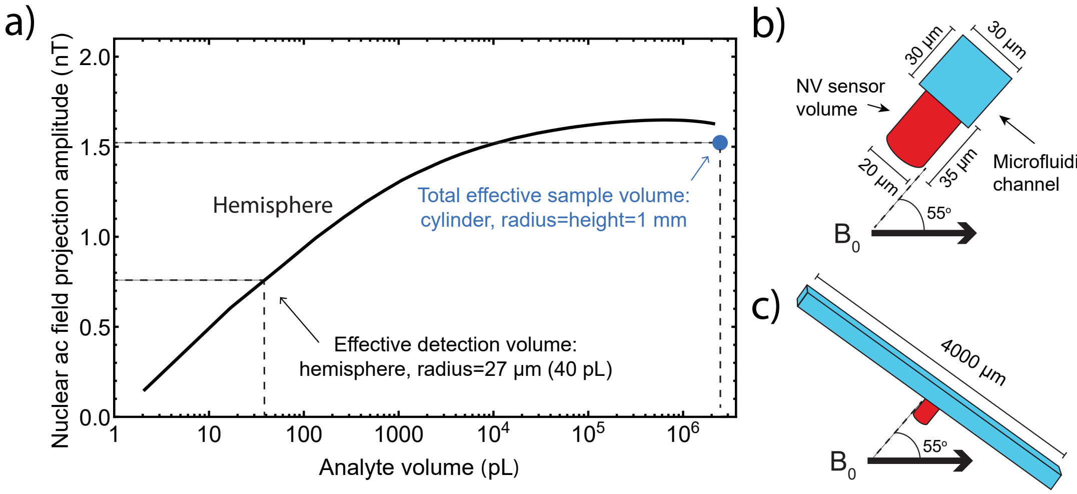

Fig. S6. Nuclear ac magnetic field projection amplitude (integrated across the sensor volume) as a function of water volume. The effective detection volume (∼40 pL) is defined as the volume where the nuclear ac field projection amplitude is equal to half of that from the total effective sample volume

For the total effective sample volume, we find that the component of the nuclear ac magnetic field amplitude along the NV axis is 1.52 nT for full nuclear spin polarization (5.1×10^{-6}). In Fig. 2(b) of the main text, we observe a nuclear ac field projection amplitude of 1.21 nT. This is ∼80% of the maximum field anticipated from total effective sample volume (1.52 nT), indicating that an effective polarization of $\sim4\times10^{-6}$ is detected.

Future microfluidic applications may call for significantly narrower channels than those studied here. It is reasonable to wonder how the nuclear ac field projection amplitude produced by the mm-sized cylindrical total effective sample volume studied here compares to those produced by the long, narrow channels used in many microfluidic applications. To study this, we modeled two microfluidic channel geometries illustrated in Fig. S6(b,c). In both cases the channels have a 30×30 μm^2 cross section and a length of 4 mm. The only difference is that in the case of Fig. S6(b) the long dimension (presumably the direction of flow) is orthogonal to B_0, whereas in Fig. S6(c), it is aligned 55° with respect to B_0. We find that the corresponding nuclear ac field projection amplitude for the first case is 0.86 nT, more than half as large as the one produced by the total effective sample volume used in our experiment. Thus, we expect our experimental geometry is a reasonable model for future miniature microfluidic channels. However in the latter case [Fig. S6(c)], the nuclear ac field projection amplitude is smaller, 0.43 nT, indicating that the orientation of the channel with respect to the experimental geometry is an important consideration in designing future microfluidic chips.

Section SXIII. Analytical calculation for heteronuclear COSY

To gain intuition for the features present in the heteronuclear COSY spectra in Fig. 4(e,f), we performed an analytical calculation on a simplified two-spin system with spin angular momenta $I_1 = 1/2$ and $I_2 = 1/2$ [36]. Note that, in our case, I_1 and I_2 correspond to nuclei of different isotopes, e.g. ^{1}H and ^{19}F. A magnetic field is applied along the z direction, and the spin Hamiltonian is:

$$\mathcal{H}/\hbar = I_{z1}\omega_1 + I_{z2}\omega_2 + 2\pi J_{12}\vec{I_1}\cdot\vec{I_2}, \tag{S6}$$

where ω_1 is the spin precession angular frequency of spin 1 and ω_2 is that of spin 2. As $|\omega_1 - \omega_2| \gg 2\pi J_{12}$ we can apply the secular approximation:

$$\mathcal{H}/\hbar = I_{z1}\omega_1 + I_{z2}\omega_2 + 2\pi J_{12} I_{z1} I_{z2}. \tag{S7}$$

We assume the spins are initially polarized along the magnetic field, resulting in an initial state:

$$I_{z1} + I_{z2}. \tag{S8}$$

We now consider the heteronuclear COSY pulse sequence beginning with a $(\pi/2)_x$ pulse acting on spin 1, followed by an evolution time t_1, and a second $(\pi/2)_x$ pulse acting on spin 2. Immediately following the first $(\pi/2)_x$ pulse acting on spin 1, the state is:

$$I_{y1} + I_{z2}. \tag{S9}$$

The evolution under the J-coupling and Zeeman terms can be applied sequentially, as both operators commute. The state evolves under J-coupling as:

$$\cos(\pi J t_1) I_{y1} - 2\sin(\pi J t_1) I_{x1} I_{z2} + I_{z2}. \tag{S10}$$

This state evolves in the external field as:

$$\begin{aligned} I_{z2} + \cos(\pi J t_1)\left[\cos(\omega_1 t_1) I_{y1} + \sin(\omega_1 t_1) I_{x1}\right] \\ -2\sin(\pi J t_1)\left[\cos(\omega_1 t_1) I_{x1} I_{z2} - \sin(\omega_1 t_1) I_{y1} I_{z2}\right]. \end{aligned} \tag{S11}$$

Next, we apply the last $(\pi/2)_x$ pulse on spin 2 and the state becomes:

$$\begin{aligned} \cancel{I_{y2}} + \cos(\pi J t_1)\left[\cos(\omega_1 t_1) I_{y1} + \sin(\omega_1 t_1) I_{x1}\right] \\ -2\sin(\pi J t_1)\left[\cancel{\cos(\omega_1 t_1) I_{x1} I_{y2}} - \cancel{\sin(\omega_1 t_1) I_{y1} I_{y2}}\right], \end{aligned} \tag{S12}$$

Here the slashes over terms show the terms that we can drop because they have no time dependence during the evolution period t_1 or they have a coherence order other than $p = \pm 1$.

To understand how the state evolves during the second evolution period we again apply the J-coupling and Zeeman evolution operators on the observable states. Evolution under J-coupling leaves the state as:

$$\begin{aligned} \cos(\pi J t_1)\Big[\cos(\omega_1 t_1)\Big\{\cos(2\pi J t_2) I_{y1} - \cancel{2\sin(2\pi J t_2) I_{x1} I_{z2}}\Big\} \\ + \sin(\omega_1 t_1)\Big\{\cos(2\pi J t_2) I_{x1} + \cancel{2\sin(2\pi J t_2) I_{y1} I_{z2}}\Big\}\Big]. \end{aligned} \tag{S13}$$

Here we have eliminated the antiphase terms which do not contribute meaningfully to the spectrum. Finally, evolution under the Zeeman interaction leaves the state as:

$$\begin{aligned} \cos(\pi J t_1)\cos(\pi J t_2)\cos(\omega_1 t_1)\left[\cos(\omega_1 t_2) I_{y1} + \sin(\omega_1 t_2) I_{x1}\right] \\ + \cos(\pi J t_1)\cos(\pi J t_2)\sin(\omega_1 t_1)\left[\cos(\omega_1 t_2) I_{x1} - \sin(\omega_1 t_2) I_{y1}\right]. \end{aligned} \tag{S14}$$

Gathering terms by spin operators, we obtain:

$$\begin{aligned} \cos(\pi J t_1)\cos(\pi J t_2)\left\{\left(\cos(\omega_1 t_1)\cos(\omega_1 t_2) - \sin(\omega_1 t_1)\sin(\omega_1 t_2)\right) I_{y1} \right. \\ \left. + \left(\cos(\omega_1 t_1)\sin(\omega_1 t_2) + \sin(\omega_1 t_1)\cos(\omega_1 t_2)\right) I_{x1}\right\} \end{aligned} \tag{S15}$$

Evidently all terms are modulated by $\cos(\pi J t_1)\cos(\pi J t_2)$. This is what leads to the off diagonal terms in the heteronuclear COSY experiments of Fig. 4(e,f).

Section SXIV. 2D homonuclear COSY of TMP

A 2D homonuclear COSY spectra was also obtained for trimethyl-phosphate, Fig. S7. We used 16 values of t_1 in 0.021 s increments up to 0.336 s. The total acquisition time was 12 hours. The presence of two diagonal peaks indicates that magnetization is modulated at the J-coupling frequency during the t_1 evolution, but no magnetization is transferred between nuclei. Like for DFB, this is expected since there is no difference in chemical shift between protons.

Section SXV. SPINACH simulations and windowing functions for 2D NMR

The 2D NMR simulations were performed using the SPINACH simulation package [47]. The relaxation rate was set to 0 Hz. The J-couplings used in the simulation are listed in table S1. The o, m and p suffixes indicate whether the two nuclei are in an ortho-, meta- or para- configuration relative to one another. Proton chemical shifts were assumed to be equal to zero.

Lorentz-to-Gauss transformations were applied to both the experimental and simulated DFB COSY spectra using the windowing function:

$$\begin{aligned} &W(t_1, t_2) = e^{\alpha_1 t_1} e^{-(\beta_1 t_1)^2} e^{\alpha_2 t_2} e^{-(\beta_2 t_2)^2}, \\ &\alpha_1 \geq 0, \beta_1 \geq 0, \alpha_2 \geq 0, \beta_2 \geq 0. \end{aligned} \tag{S16}$$

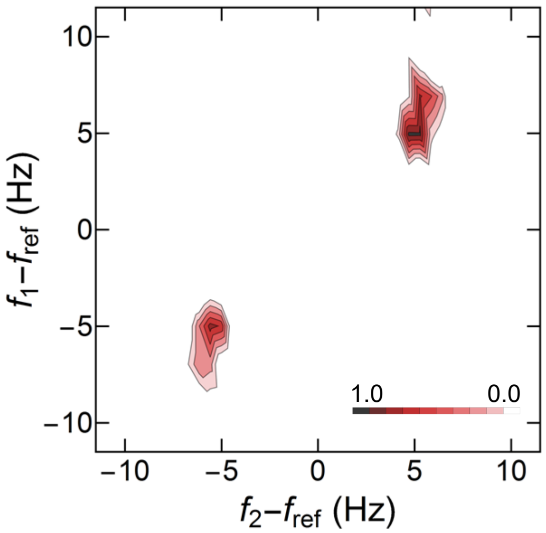

Fig. S7. Experimental homonuclear COSY spectrum of TMP. The normalized absolute value of the 2D Fourier Transform is plotted.

Table S1. Values of the different *J*-couplings in a DFB molecule used in the simulation.

Type of coupling	Coupling strength
$J^o[^{19}F,^1H]$	7.6 Hz
$J^m[^{19}F,^1H]$	4.6 Hz
$J^o[^1H,^1H]$	8 Hz
$J^m[^1H,^1H]$	2 Hz
$J^p[^1H,^1H]$	0 Hz
$J^p[^{19}F,^{19}F]$	12 Hz

The same $\alpha_{1,2}$ and $\beta_{1,2}$ parameters were used for the experimental and simulated spectra. For the heteronuclear dataset, we used $\alpha_{1,2} = 1.2$ s^{-1} and $\beta_{1,2} = 4$ s^{-1}. This windowing process contributed ∼3 Hz to the width of the NMR lines in both the f_1 and f_2 dimensions. In the homonuclear dataset the parameters were $\alpha_1 = 3$ s^{-1}, $\alpha_2 = 1.6$ s^{-1}, $\beta_1 = 2.8$ s^{-1}, and $\beta_2 = 2.5$ s^{-1}, which contributed ∼2.2 Hz to the width of the NMR lines in both f_1 and f_2 dimensions.
